# Lowering Hippocampal miR-29a Expression Slows Cognitive Decline and Reduces Beta-Amyloid Deposition in 5×FAD Mice

**DOI:** 10.1007/s12035-023-03791-0

**Published:** 2023-11-22

**Authors:** Zhen Mei, Jiaqi Liu, Jason P Schroeder, David Weinshenker, Duc M. Duong, Nicholas T. Seyfried, Yujing Li, Peng Jin, Aliza P. Wingo, Thomas S. Wingo

**Affiliations:** 1 Department of Neurology, Emory University School of Medicine, Atlanta, GA USA; 2 Present Address: Department of Neurology, Affiliated Hospital of Guizhou Medical University, Guiyang, China; 3 Department of Human Genetics, Emory University School of Medicine, Atlanta, GA USA; 4 Goizueta Alzheimer’s Disease Research Center, Emory University School of Medicine, Atlanta, GA USA; 5 Department of Biochemistry, Emory University School of Medicine, Atlanta, GA USA; 6 Division of Mental Health, Atlanta VA Medical Center, Decatur, GA USA; 7 Department of Psychiatry, Emory University School of Medicine, Atlanta, GA USA

**Keywords:** miR-29a, Cognition, Beta-amyloid, Neuroinflammation, Wdfy1

## Abstract

microRNA-29a (miR-29a) increases with age in humans and mice, and, in the brain, it has a role in neuronal maturation and response to inflammation. We previously found higher miR-29a levels in the human brain to be associated with faster antemortem cognitive decline, suggesting that lowering miR-29a levels could ameliorate memory impairment in the 5×FAD AD mouse model. To test this, we generated an adeno-associated virus (AAV) expressing GFP and a miR-29a “sponge” or empty vector. We found that the AAV expressing miR-29a sponge functionally reduced miR-29a levels and improved measures of memory in the Morris water maze and fear condition paradigms when delivered to the hippocampi of 5×FAD and WT mice. miR-29a sponge significantly reduced hippocampal beta-amyloid deposition in 5×FAD mice and lowered astrocyte and microglia activation in both 5×FAD and WT mice. Using transcriptomic and proteomic sequencing, we identified Plxna1 and Wdfy1 as putative effectors at the transcript and protein level in WT and 5×FAD mice, respectively. These data indicate that lower miR-29a levels mitigate cognitive decline, making miR-29a and its target genes worth further evaluation as targets to mitigate Alzheimer’s disease (AD).

**Supplementary Information:**

The online version contains supplementary material available at 10.1007/s12035-023-03791-0.

## Introduction

Alzheimer’s disease (AD) is the most common cause of dementia. Recent epidemiological data indicate that the number of people with AD worldwide will increase to 131.5 million by 2050 [1]. AD is a neurodegenerative disorder characterized by progressive cognitive decline, neuronal loss, and brain pathology. Its neuropathological hallmarks are neuritic plaques comprised of amyloid-β aggregates and tau-containing neurofibrillary tangles. The pathologic hallmarks of AD typically coexist with other age-related pathologies (e.g., Lewy bodies and vascular disease), which are common in people over 70 years and collectively account for only about 40% of the cognitive decline [2, 3]. This observation has led to investigations of other potential contributing factors, including the microRNAs (miRNAs) [4, 5].

miRNAs are small non-coding RNAs that regulate gene expression by either mRNA degradation or translation inhibition [6]. Each miRNA can target hundreds of transcripts, thereby potentially exerting a widespread influence on the transcriptional landscape of a cell. Certain miRNAs are implicated in synaptic plasticity, neuronal survival, and aggregation of neurodegenerative pathologies [7–9]. Our previous work identified miRNAs associated with cognitive trajectory using postmortem brain specimens in a global miRNA association study [5]. Among these, miR-132 and miR-29a were the most significantly associated miRNA with cognitive trajectory, even after accounting for the eight measured age-related brain pathologies [5]. The role of miR-132 in aging and AD has been well-documented in other studies [4, 10–13]. miR-29a has been less studied and its role in AD is not well understood. Because higher miR-29a level was associated with faster cognitive decline [5], we hypothesized that lowering miR-29a levels would improve cognitive performance.

Here we aimed to test our hypothesis that reducing miR-29a would slow cognitive decline using 5×FAD transgenic mice and their wild-type (WT) littermates and to investigate the downstream effects of lowering miR-29a at the transcript and protein level. We first designed a miR-29a sponge to “soak up” the microribonucleoprotein complexes (microRNPs) loaded with miR-29a and thereby derepress its downstream targets. AAV expressing miR-29a sponge or control AAV were stereotaxically injected into the hippocampi of 5×FAD and WT mice. Cognition was assessed by the Morris water maze and fear conditioning; amyloid deposition and activated astrocytes and microglia were evaluated by immunofluorescence staining, and RNA-sequencing and deep proteomic sequencing were performed to identify the downstream effectors of miR-29a. We found that miR-29a’s loss-of-function improved learning and memory and attenuated amyloid deposition and immune cell activation in 5×FAD and WT mice. Transcriptomic and proteomic analyses identified targets of miR-29a and suggested a role for miR-29a in modulating neuroinflammation.

## Methods

### Mice

Female 5×FAD and C57BL/6 mice used in the studies were group-housed (maximum of 5 animals per cage) in the Department of Animal Resources at Emory University under standard conditions. 5×FAD transgenic mice (The Jackson Laboratory, #034848) were purchased and maintained as hemizygotes on a C57BL/6 background. 5×FAD transgenic mice were confirmed by polymerase chain reaction and non-transgenic WT littermates were used as controls. All experiments were conducted in strict accordance with the National Institutes of Health Guide for the Care and Use of Laboratory Animals and approved by the Emory Institutional Animal Care and Use Committee.

### Construction of miR-29a Mimic

miR-29a mimic was constructed by first amplifying fragment-encoding pre-miR-29a from the genomic DNA isolated from HEK-293T cells. The PCR product was then purified, digested with EcoRI and XbaI, and inserted into a digested pAAV-MCS vector (Addgene, VPK-410). The construct was verified by Sanger sequencing. The primer sequences for miR-29a mimic are given in Supplementary Table 1.

### miR-29a Sponge Design and Cloning

miR-29a sponge was seven tandemly arrayed miR-29a binding sites separated by a 4-nt spacer, each of which was perfectly complementary in the seed region but with a bulge at positions 9–12 to prevent degradation by Argonaute 2 [14]. We annealed, ligated, gel purified, and cloned miR-29a sponge into 3′UTR of psiCHECK-2 vector (Promega, C8021) and pAAV-GFP vector (Cell biolabs, AAV-400), respectively. All constructs were verified by Sanger sequencing. The primer sequences for miR-29a are given in Supplementary Table 1.

### Luciferase Assays

We plated HEK-293T cells into 24-well plates the day before transfection and transfected them in triplicate with psiCHECK-2 vector containing miR-29a sponge together with miR-29a mimic or empty vector (pAAV-MCS) at a ratio of 1:20 (300 ng total DNA/well). Lipofectamine 2000 (Invitrogen, 11668019) was used as the transfection reagent. Cells were lysed in a passive lysis buffer 48 h after transfection and assayed in triplicate using the Dual-Luciferase Reporter Assay System (Promega, E1910). Renilla luciferase activity was normalized to Firefly luciferase activity measured on a GloMax 96 microplate luminometer (Promega, E6521) and was then calculated relative to the negative control in each independent replicate.

### Western Blots

HEK-293T cells were plated into 6-well plates the day before transfection. miR-29a mimic together with pAAV-GFP vector containing miR-29a Sponge or empty vector (pAAV-GFP) were co-transfected into HEK-293T cells at a ratio of 20:1 with lipofectamine 2000 (1000 ng total DNA/well). Fluorescence microscopy was used to check the percentage of GFP-positive cells 24 h after transfection. At 48 h after transfection, cells were lysed in RIPA buffer (Thermo Fisher Scientific, 89900) supplemented with Protease Inhibitor Cocktail (Roche, 4693159001). Protein concentrations were determined with the BCA Protein Assay kit (Thermo Scientific, 23235) according to the manufacturer’s instructions. Equal amounts of total proteins were resolved on a 10% Mini-Protean TGX precast gel (Bio-Rad, 4561033), and transferred to a PVDF membrane (Bio-Rad,1704156) with the Trans-Blot Turbo transfer system (Bio-Rad). The membranes were incubated with a blocking solution (5% skim milk in phosphate-buffered saline with 1% Tween-20) for 1 h, blotted by a blocking solution containing a primary antibody (DNMT3A, Cell Signaling Technology, #3598, 1:1000; GAPDH, Invitrogen, #39-8600, 1:10000) overnight at 4 °C, and incubated with a horseradish peroxidase-conjugated secondary antibody for 1 h. DNMT3A is a well-validated target of miR-29 [15–17]. Proteins were visualized by ECL prime detection reagent (Cytiva, RPN2232) and ChemiDoc imaging system (Bio-Rad). Immunoreactive bands were quantified with ImageJ software. The experiment was performed in triplicate.

### Adeno-associated Virus (AAV) Production and Stereotaxic Injection

The plasmid templates for AAV generation were pAAV-GFP vector containing miR-29a sponge or the empty vector. AAV9 viruses (AAV-miR-29a sponge and control AAV) were generated by Emory University Viral Core. Before AAV injection, female 5×FAD and WT mice were anesthetized and placed in a stereotaxic frame. After a skin incision was made, holes were drilled at *x* (± 1.5 mm from bregma) and *y* (−2.0 mm from bregma). AAV-miR-29a sponge or control AAV were injected into the left and right hippocampi (*z* = −1.9 mm from bregma) respectively, with 6.2 × 10^10^ total viral particles per side and delivered at a rate of 0.2 μL/min. The syringe was left in place for 5 min and withdrawn slowly after each injection. When the injection was complete, the skin was sutured and sterilized.

### Morris Water Maze

Experiments were conducted by the Emory University Rodent Behavioral Core by trained personnel who were blinded to the mouse condition. In a circular 52-inch-diameter tank filled with opaque water kept at 23 °C with a hidden circular platform (30 cm diameter) present 1 cm below the water in the northwestern quadrant of the tank, each mouse had four training trials to find the platform per day over 5 consecutive days. Each training trial lasted a maximum of 60 s. If a mouse did not find the platform in time, it was manually guided to it and placed on the platform for 10 s. Escape latency to the platform as well as swim speed were recorded by an automated tracking system (TopScan, CleverSys). A probe trial was conducted on the sixth day where the platform was removed, and the mice were released from the south start point and allowed to swim for 60 s. The tracking system recorded the percentage of search time in the quadrant where the platform was previously located.

### Fear Conditioning

Fear conditioning was conducted by the Emory University Rodent Behavioral Core by trained personnel who were blinded to the mouse condition. Fear conditioning occurred over 3 consecutive days in a chamber (H10-11M-TC, Coulbourn Instruments) equipped with a house light, a speaker, a ceiling-mounted camera, and an electric grid shock floor that could be replaced with a non-shock wire mesh floor. Fear conditioning training on day 1 began with a 3-min acclimatization period followed by 3 tone-shock pairings during which the tone lasted 20 s and was co-terminated with a 3-s, 0.5-mA foot shock. Mouse behavior was recorded for 60 s after a tone-shock pairing before the next round. Contextual fear testing on day 2 was conducted in the same chamber as day 1 without any tone or shock. Cued fear testing on day 3 was conducted in a different chamber with a non-shock wire mesh floor and began with a 3-min acclimatization period followed by a 5-min tone without any shock. FreezeFrame software (Coulbourn Instruments) was used to record freezing behavior and the percentage of freezing time was determined.

### RNA Isolation and Real-Time Quantitative PCR

Samples were homogenized in TRIzol reagent (Thermo Fisher Scientific, 15596018) and shaken for 15 s after the addition of chloroform. The samples were transferred to pre-spun Phase Lock Gel-Heavy tubes (Quanta bio, 2302830) and incubated at RT for 5 min and then centrifuged at 12,000 g/4 °C for 15 min. The upper phase aqueous solution was collected in a fresh tube and RNA was precipitated by isopropanol. Samples were gently mixed and left at −80 °C overnight and then centrifuged at 14,000 rpm/4 °C for 25 min. RNA pellet was washed twice in 75% ethanol and resuspended in nuclease-free water. miR-29a was reverse transcribed to cDNA using miR-29a-specific primers from TaqMan (Thermo Fisher Scientific, Assay ID: 002112). Real-time polymerase chain reaction was performed using the Applied Biosystems TaqMan Gene Expression assay following the manufacturer’s instruction. Data were analyzed by the △△Ct methods using U6 as an endogenous control.

### Tissue Preparation

After cervical dislocation, mouse brains were removed and dissected at the midline. For biochemical analysis, hippocampi were dissociated and immediately snap-frozen in liquid nitrogen and stored at −80 °C for protein and RNA sequencing. For immunofluorescence staining, mice were anesthetized and transcardially perfused with 0.9% sodium chloride and then fixed in ice-cold 4% paraformaldehyde in 1 × PBS. The brains were removed and postfixed in 4% paraformaldehyde overnight at 4 °C and transferred to 30% sucrose at 4 °C for 48 h before being embedded for cryostat sectioning.

### Immunofluorescence Staining

Mouse brains were embedded with optimal cutting temperature compound (Tissue-Tek, 4583) and cut into serial 10-μm-thick coronal sections with a cryostat (Leica Biosystems). The sections were washed three times in 1 × PBS for 5 min each and incubated with blocking buffer (PBS with 10% normal goat serum and 0.25% Triton X-100) for 1 h at RT. The sections were then incubated with primary antibodies overnight at 4 °C, followed by incubation with Alexa-fluorophore-conjugated secondary antibodies (Thermo Fisher Scientific, A-11004, A-11011, 1:500) for 1 h at RT in the dark. Sections were rinsed and mounted onto slides using Vectashield mounting medium with DAPI (Vector Laboratories, H-1200). The following primary antibodies were used for immunofluorescence staining: anti-beta amyloid (Abcam, ab2539, 1:200); anti-Iba1 (Wako Chemicals, 019-19741, 1:200); and anti-GFAP (Abcam, ab7260, 1:200). Immunoreactivity (IR) was calculated as mean gray value (area of IR within ROI divided by total area of ROI) within ImageJ as previously described [18]. Images were obtained on a ZEISS 710 confocal laser-scanning microscope.

### RNA Sequencing

The total RNA used in the sequencing study was isolated from the hippocampus using TRIzol reagent (Thermo Fisher Scientific, 15596018). RNA quality was measured on an Agilent 2100 Bioanalyzer system based on the 28S/18S ratio and the RNA integrity number (RIN). For each sample, 1 μg RNA was used to construct sequencing libraries using Illumina’s TruSeq RNA Sample Prep Kit. Samples were sequenced on Illumina’s HiSeq 2000 system with a sequencing depth of 40 M total reads per sample (20 M for each direction), producing sequencing results in FastQ format. The QC on the sequence reads was done with FastQC, and all samples were carried forward in the analysis. Sequenced data were aligned to a mouse reference genome using STAR aligner version 2.7.3a [19] and STAR produces a read count file for each sample using the algorithm of htseq-count [20] with default settings.

### Gene Differential Expression Analysis

The differential expression analysis across experiment and control groups was implemented in R (version 4.1.2). Variance stabilizing transformation (VST) function offered by DESeq2 R package (version 1.32.0) [21] was used to log_2_ transform the raw counts, normalize for library sizes, and reduce heteroskedasticity. The Surrogate Variable Analysis (SVA) method in the sva R package (version 3.40.0) [22] was used to detect potentially hidden variables from the normalized data. Given our sample size is small (*n* = 8), we only included the first surrogate variable (SV1) in the design matrix [22]. The R package DESeq2 was used to perform the differential expression analysis adjusted for SV1. The Benjamini-Hochberg method was used to control for the false discovery rate (FDR) and was considered significant at FDR less than 0.1. The R package clusterProfiler [23] (version 4.0.5) was used to perform gene ontology enrichment analysis for the significant genes following instructions in the package vignette. GO terms were considered significant at an FDR-adjusted *p*-value of less than 0.05.

### Proteomic Sequencing

Each mouse hippocampus sample was digested individually using the EasyPep™ mini sample preparation kit according to manufacturer instructions (ThermoFisher Scientific, A40006). The resulting peptides were reconstituted in 100 ul of 100 mM triethyl ammonium bicarbonate (TEAB) and labeling was performed as previously described [24, 25]. High pH fractionation was performed essentially as previously described [26]. Liquid chromatography-tandem mass spectrometry followed by protein identification and quantification were performed as described in detail in supplementary methods.

### Protein Differential Expression Analysis

The normalization and differential analysis of the proteomics data were performed in R (version 4.1.2). We included proteins with TMT abundance values in at least 50% replicates per group and displayed a high protein FDR confidence (FDR < 0.01). To normalize the raw data, for each sample, each protein’s abundance was first divided by the sum of abundance values of all the proteins profiled for that sample, followed by the log2 transformation. For each protein, we then constructed a linear model of normalized abundance as a function of the group. The *p*-values were adjusted for multiple comparisons using the FDR method. We next selected proteins that are predicted targets of miR-29a based on the miRDB [27, 28] database and then performed the differential expression analysis. Proteins were declared to be significant at an FDR-adjusted *p*-value of less than 0.1 using the Benjamini–Hochberg control for the FDR.

### Experimental Design and Statistical Analysis

For AAV injection, female mice at 6–7 months of age (WT or 5×FAD) were injected with a control AAV or AAV-miR-29a sponge. For simplicity, we refer to the injection groups as WT-Control, WT-Sponge, FAD-Control, and FAD-Sponge. The number of mice in each group was WT-Control (*n* = 12), WT-Sponge (*n* = 11), FAD-Control (*n* = 9), and FAD-Sponge (*n* = 10). The behavioral tests were performed 3 months after injection. We then randomly selected 3 mice from each group for immunofluorescence staining. For RNA-Seq and proteomics analyses, 4 mice were randomly selected from each group, and one side of the hippocampus was harvested for RNA-Seq analysis and the other side was for proteomics.

The expression of miR-29a and behavioral testing (i.e., Morris water maze and fear conditioning) were tested using two-way ANOVA or repeated measures (RM) ANOVA, followed by *post hoc* methods to control for multiple comparisons. Values were considered significant at *p* < 0.05 and a tendency at *p* <0.1. Calculations were performed and figures were created using Prism version 8.3 for Windows.

## Results

### Endogenous Expression Level of miR-29a in Mouse Hippocampus

**Fig. 1:**
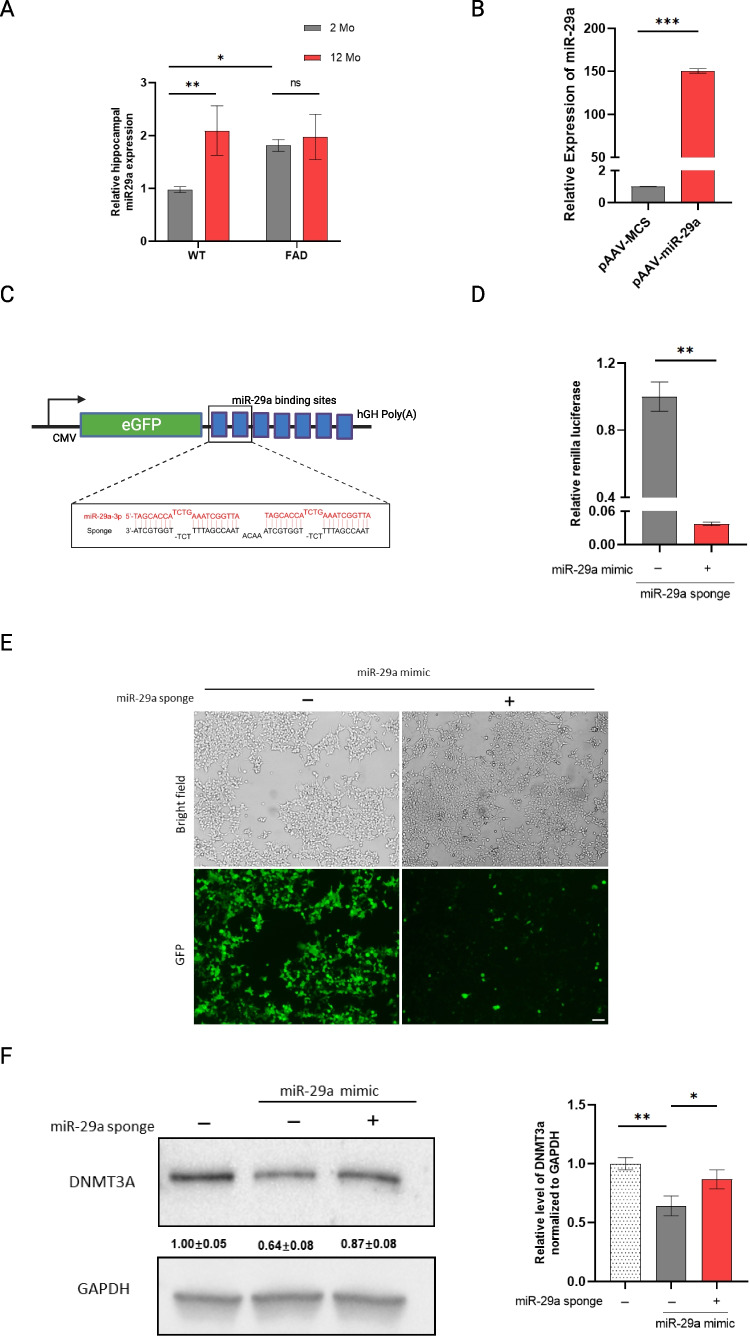
miR-29a expression in mouse hippocampus and miR-29a sponge validation in vitro. **A** miR-29a was determined in the hippocampus of 2- and 12-month-old 5×FAD and WT mice by real-time RT-PCR. U6 snRNA was used as the endogenous control. Data was analyzed via two-way ANOVA (age by genotype) with Sidak's post hoc tests. Bars indicate mean ± standard deviation (SD) with *n* = 3 per group; NS not significant; *p < 0.05, **p < 0.01. **B** Relative expression level of mature miR-29a in HEK-293T cells transfected with miR-29a mimic (pAAV-miR-29a) and control vector (pAAV-MCS); ***p < 0.001. **C** Schematic representation of miR-29a sponge mechanism and design. Sponge sequence was shown as black color and the target miRNA was shown as red. **D** Renilla luciferase activity was assayed relative to firefly luciferase activity in 293T cells transfected with psiCHECK2 vector containing miR-29a sponge and miR-29a mimic (pAAV-miR-29a) or control vector (pAAV-MCS); **p < 0.01. **E** Cell morphology and GFP expression upon transfection of miR-29a mimic (pAAV-miR-29a) with pAAV-GFP vector containing miR-29a sponge or empty vector (pAAV-GFP), as observed by light and fluorescent microscopy. Scale bars represent 100 μm. **F** Western blot analysis of DNMT3A upon transfection of miR-29a mimic with or without miR-29a sponge. Experiments were performed 3 times and one representative western blot is shown. The quantification of western blots is provided. Data represents fold change relative to no treatment group ± SD. GAPDH was used as a loading control. *p < 0.05, **p < 0.01

The endogenous expression of miR-29a was measured in the hippocampi of young (2-month-old) and aged (12-month-old) 5×FAD and WT mice, respectively (Fig. 1A). Two-way ANOVA revealed there was a main effect of age (*F*_(1, 8)_ = 11.66, *p* = 0.0092) and an age × genotype interaction (*F*_(1, 8)_ = 6.531, *p* = 0.0339). *Post hoc* Sidak’s tests showed that the 12-month-old WT mice had significantly higher levels of miR-29a than the 2-month-old WT (*t*_(8)_ = 4.221, *p* = 0.0058), while there was no significant difference between young and aged 5×FAD mice. Within genotype, *post hoc* Šidák correction showed that the 2-month-old 5×FAD displayed higher miR-29a than 2-month-old WT mice (*t*_(8)_ = 3.164, *p*= 0.0265), while there was no difference between 5×FAD and WT mice at 12-months. These data suggested miR-29a increased with age specifically in WT mice and miR-29a displayed a higher expression level in young 5×FAD mice.

### Testing Efficacy of the miR-29a Sponge

The overexpression of miR-29a by the miR-29a mimic was confirmed in HEK-293T cells (Fig. 1B). Yet, for this study, we focused on miRNA loss-of-function to study the role of miR-29a because it reveals miRNA functions that depend on physiological levels, while miRNA overexpression can result in repression of non-physiological mRNA targets [29]. Thus, we constructed a miR-29a sponge with seven tandemly arrayed miR-29a binding sites to functionally downregulate the level of miR-29a (Fig. 1C). To investigate the efficacy of the miR-29a sponge, we cloned miR-29a sponge into the 3′UTR of the Renilla luciferase gene of the psiCHECK2 vector and co-transfected the miR-29a sponge with or without miR-29a mimic in HEK-293T cells (Fig. 1D). As expected, we observed a decreased Renilla to Firefly ratio upon transfection with miR-29a sponge and miR-29a mimic (Fig. 1D), confirming the effectiveness of the sponge transcript binding to the miR-29a.

To test the ability of the miR-29a sponge to derepress downstream targets, we next assayed the protein Dnmt3a, a well-validated target of miR-29 [15–17]. We cloned the miR-29a sponge into the 3′UTR of the GFP gene of the pAAV-GFP vector. miR-29a mimic with or without the sponge construct was then co-transfected HEK-293T cells and cells were observed for GFP expression at 24 h using light and fluorescence microscopy. Figure 1E shows that GFP expression of the construct containing miR-29a sponge was visibly repressed upon transfection with miR-29a mimic relative to the GFP control construct. We harvested the cells at 48 h and assayed the protein Dnmt3a by Western blot. As shown in Fig. 1F, Dnmt3a expression decreased by about 40% upon miR-29a overexpression and miR-29a sponge rescued the target by about 20%. These results further confirmed miR-29a sponge acted as a competitive regulator that sequestered miR-29a and prevented miRNA/mRNA interaction.

### Testing Effect of miR-29a Sponge in Mouse Hippocampi on Learning and Memory

**Fig. 2:**
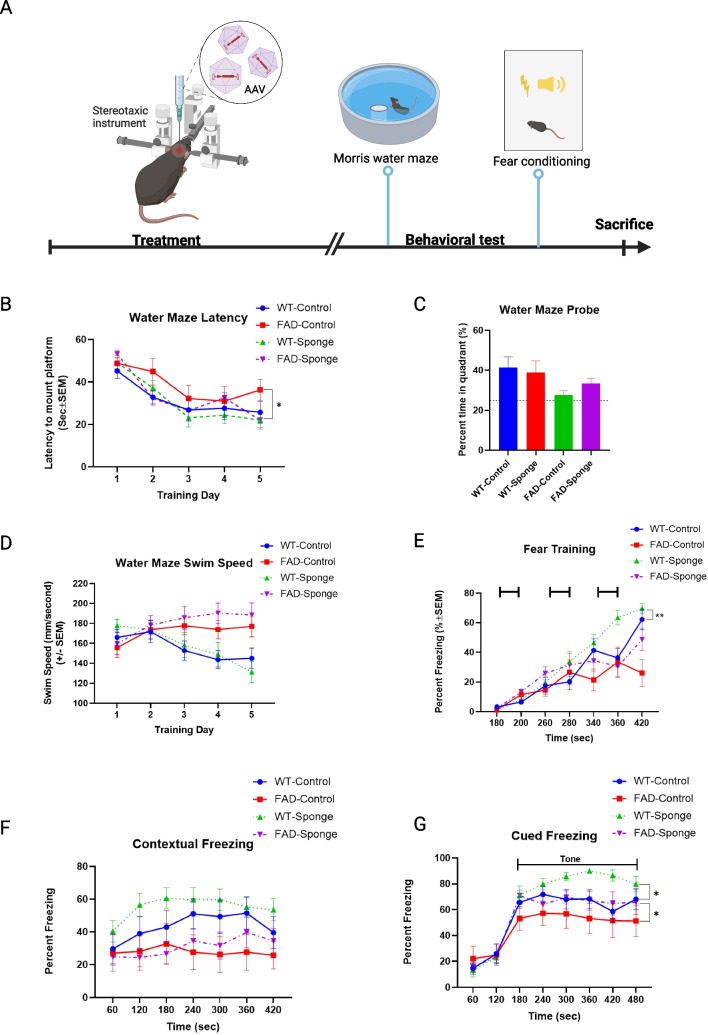
Results of Morris water maze and fear conditioning. **A** Timeline of experiments. **B** Latency to find the platform. **C** Water maze probe trial. **D** Swim speed during 5 days of training trial. **E** Fear training with 3 tone-shock pairings. **F–G** Percentage of time spent in freezing following re-exposure to the shock-associated context or tone. Data are mean ± SEM with *n* = 9–12 per group. Data were analyzed via two-way repeated measures ANOVA (time × group) or three-way repeated measures ANOVA (time × group × genotype). The water maze probe trial was analyzed via two-way ANOVA (genotype by group). *p < 0.05, **p < 0.01 based on post hoc pairwise comparisons

To determine whether functionally downregulating the level of miR-29a impacted cognitive performance, we injected control AAV or AAV-miR-29a sponge into the hippocampi of 5×FAD and WT mice, respectively. The potential effect of derepressing miR-29a targets on learning and memory was tested using the Morris water maze and fear conditioning with the more stressful tasks performed first (Fig. 2A).

The Morris water maze is considered a hippocampal-dependent learning and memory task. Mice were first trained for 5 consecutive days to learn the location of a hidden platform, and escape latency and swim speed were recorded. In the subsequent probe trial, mice were returned to the maze without the platform and the amount of time they spent in the platform quadrant was recorded.

All groups showed progressively shorter escape latencies over the 5 training days, and FAD-sponge generally showed lower latencies compared to FAD-control (Fig. 2B). Two-way repeated measures (RM) ANOVA between FAD-Sponge and FAD-Control revealed a main effect of time (*F*_(3.596, 68.33)_ = 12.4, *p* < 0.0001) and a time × group interaction (*F*_(4, 76)_ = 2.512, *p*=0.0485). *Post hoc* Dunnett’s test showed that compared with day 1 of training, FAD-Sponge had significantly lower escape latency starting from day 2 (*t*_(10)_ = 7.136, *p*= 0.0001) and the remaining training days (day3, *t*_(10)_ = 5.112, *p* = 0.0016; day4, *t*_(10)_ = 4.598, *p* = 0.0033; and day 5, *t*_(10)_ = 6.864, *p* = 0.0002, respectively). By contrast, FAD-Control failed to demonstrate significant learning until day 4 of training (*t*_(9)_ = 3.519, *p* = 0.0208). In WT mice, there was a main effect of time (*F*_(2.468, 51.83)_ = 15.42, *p* < 0.0001), but no effect of time × group interaction. In the probe trial, all groups spent more than 25% of their time (i.e., greater than chance) swimming in the quadrant previously containing the platform, and WT mice spent significantly more time compared with 5×FAD mice (main effect of genotype: *F*_(1, 40)_ = 4.685, *p* = 0.0364). Although the FAD-Sponge mice tended to spend more time in the target quadrant than the FAD-Control mice, there were no significant effects of group or group × genotype interaction (Fig. 2C). We found no evidence for differences in swim speed between control or sponge conditions in either WT or FAD mice using a two-way ANOVA, but 5×FAD mice swam faster than WT mice (main effect of genotype: *F*_(1, 20)_ = 4.892, *p* = 0.0388, Fig. 2D). Taken together, these results indicate that downregulating the miR-29a level can ameliorate spatial learning deficits in 5×FAD mice.

We next assessed mice in fear conditioning, a hippocampal-dependent measure of associative learning and memory. During fear training, mice were placed in the fear conditioning chambers and exposed to 3 tone-shock pairings on day 1, and the percentage of freezing was recorded. The following day, mice were returned to the same chamber in the absence of tone or shock to assess contextual memory. On the third day of training, mice were placed in a novel chamber, and freezing in response to the tone alone was recorded to test cued fear memory.

During fear training, all groups showed learning in response to tone-shock pairings (Fig. 2E). RM two-way ANOVA between WT-Sponge and WT-Control revealed a main effect of time (*F*_(3.781, 79.41)_ = 68.39, *p* < 0.0001) and a significant time × group interaction (*F*_(6, 126)_ = 3.01, *p* = 0.0088). Post hoc Sidak’s test showed WT-Sponge froze significantly more at the 360-second timepoint compared with WT-Control (*t*_(20.25)_ = 3.414, *p* = 0.0189). FAD-Sponge and FAD-Control also showed more freezing with more training time (*F*_(2.938, 49.94)_ = 14.07, *p* < 0.0001), but there was no significant time × group interaction. For contextual memory, WT mice froze significantly more than 5×FAD mice (main effect of genotype: *F*_(1, 20)_ = 6.102, *p* = 0.0226), but neither WT- nor FAD-sponge showed significant differences compared with their control groups (Fig. 2F). All groups displayed cued freezing with the presentation of the tone on the third day of training (Fig. 2G). WT mice displayed more cued freezing than 5×FAD mice (main effect of genotype: *F*_(1, 20)_ = 4.462, *p* = 0.0474), and WT- and FAD-sponge groups showed significant differences compared with their control groups (main effect of group: *F*_(1, 20)_ = 4.668, *p* = 0.043). Overall, the miR-29a sponge improved cued fear memory in both WT and 5×FAD mice.

### miR-29a Sponge-Attenuated Amyloid Deposition and Gliosis

**Fig. 3:**
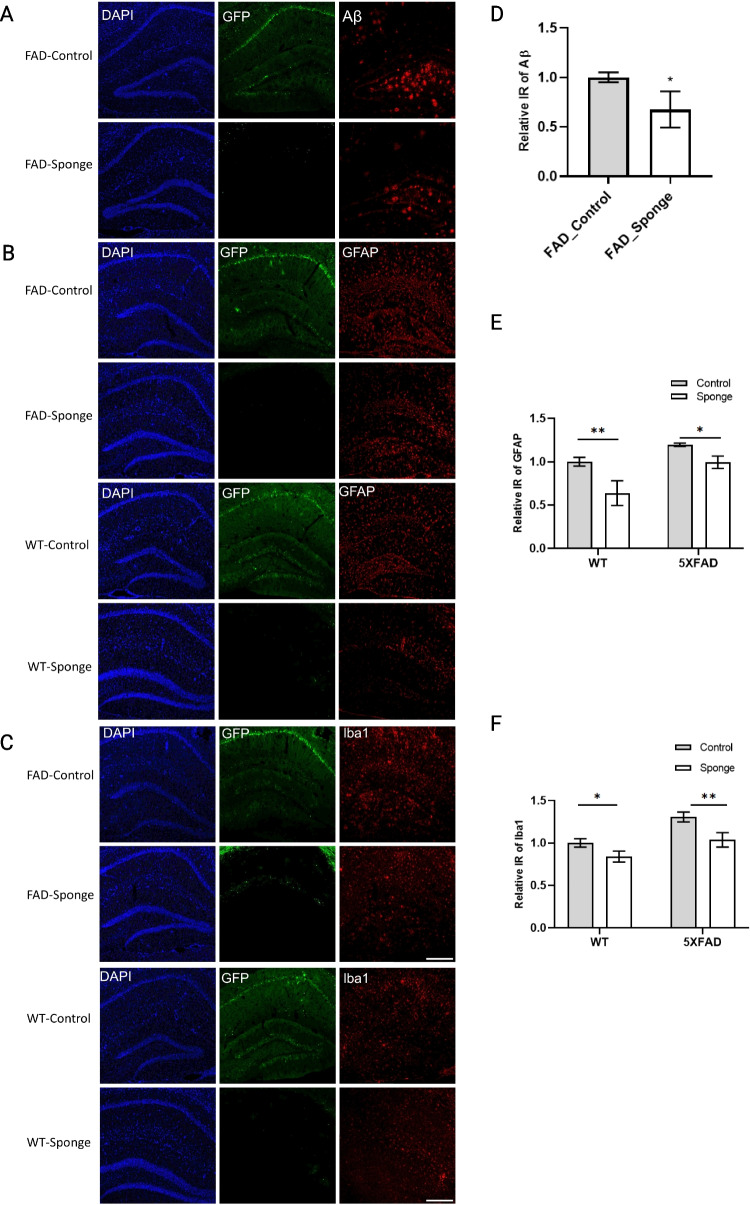
miR-29a sponge attenuates amyloid deposition and decreases activated astrocytes and microglia in the hippocampus of 5×FAD mice and WT mice. **A–C** Representative immunofluorescence images. **D–F** Quantification of Aβ, GFAP, and Iba1 immunoreactivity. Data were analyzed via two-way ANOVA (genotype by group) with Sidak’s post hoc tests. Bars indicate mean ± SD. *N* = 3 per group. Scale bars represent 100 μm; *p < 0.05, **p < 0.01, compared with the control group

To identify potential mechanisms for the amelioration of behavioral outcomes, we evaluated the hippocampi for beta-amyloid accumulation and neuroinflammation. The beta-amyloid deposition was evaluated exclusively in 5×FAD mice since WT mice do not show any of this pathology. Robust amyloid plaque deposition was in the hippocampus of FAD-Control mice, which was significantly reduced in FAD-Sponge mice (Fig. 3A and D, *t*_(4)_ = 2.983, *p* = 0.0406). Neuroinflammation was detected by the presence of markers for activated astrocytes and microglia [30]. Both FAD- and WT-sponge mice showed decreased levels of GFAP immunoreactivity compared with their control groups (5×FAD, *t*_(8)_ = 2.935, *p* = 0.0374; WT, *t*_(8)_ = 5.264, *p* = 0.0015, Fig. 3B and E). For microglia, both FAD- and WT-sponge mice showed attenuated microglial activation (5×FAD, *t*_(8)_ = 5.004, *p* = 0.0021; WT, *t*_(8)_ = 2.965, *p* = 0.0357, Fig. 3C and F). GFP expression was visibly repressed in both FAD- and WT-Sponge groups relative to their control groups, further confirming the efficacy of the miR-29a sponge. These data demonstrate that the miR-29a sponge-attenuated amyloid burden and neuroinflammation in 5×FAD and WT mice.

### Identifying Putative Targets of miR-29a at Both the Transcript and Protein Levels

To investigate the potential downstream effectors of miR-29a, we performed transcriptomic and proteomic profiling from 5×FAD and WT mouse hippocampi.

**Fig. 4:**
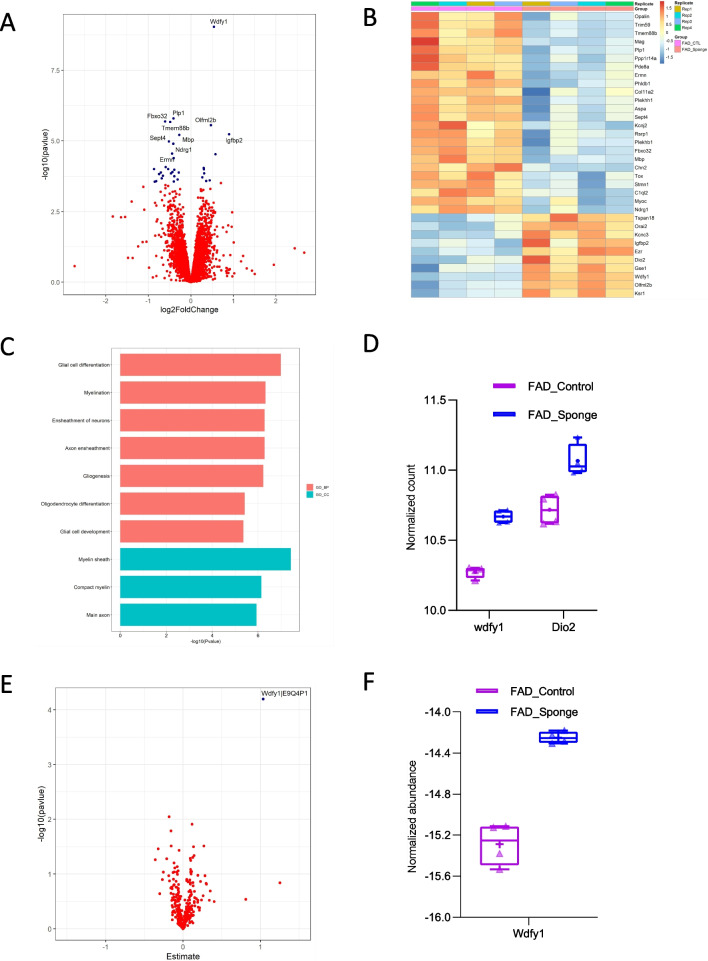
Downstream effectors of miR-29a at the transcript and protein level in 5×FAD mice. **A** Volcano plot displaying the distribution of differentially expressed genes between FAD-Sponge and FAD-Control in the RNA-Seq analysis. Top10 DEGs were labeled. **B** Heatmap of DEGs between FAD-Sponge and FAD-Control. Downregulated DEGs in FAD-Sponge vs FAD-Control were depicted in blue and upregulated DEGs were in orange. **C** GO enrichment analysis of downregulated DEGs in FAD-Sponge. **D** Expression of DEGs that are predicted targets of miR-29a. **E** Volcano plot displaying the distribution of differentially expressed proteins between FAD-Sponge and FAD-Control. **F** Expression of the differentially expressed protein between FAD-Sponge and FAD-Control

From transcriptomic profiling of eight 5×FAD and control mice, we found 34 transcripts that differed between control and miR-29a-sponge conditions at FDR < 0.1. Of the 34 DEGs, 24 were downregulated and 10 were upregulated in FAD-Sponge compared with FAD-Control (Fig. 4A and B, Supplementary Table 2). To glean biological functions of the DEGs, we performed gene ontology (GO) enrichment analysis for downregulated genes in FAD-Sponge that showed genes enriched in glial cell differentiation, myelination, ensheathment of neurons, and axon ensheathment at adjusted *p*-value < 0.05 (Fig. 4C). Since miR-29a was downregulated in FAD-Sponge, its downstream targets are expected to be upregulated. We found Wdfy1 and Dio2 were DEGs upregulated in FAD-Sponge and predicted to be targets of miR-29a [27] (Fig. 4D), supporting them as putative targets of miR-29a.

**Fig. 5:**
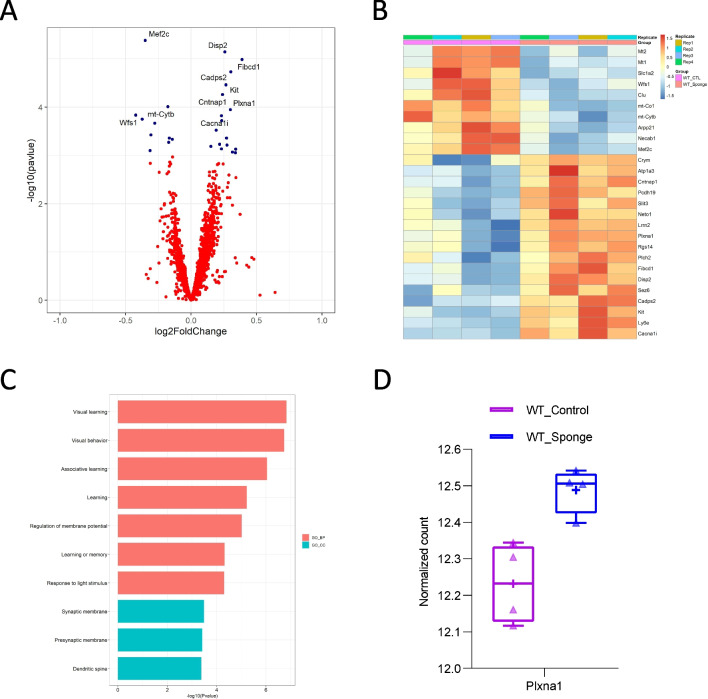
Downstream effectors of miR-29a at the transcript level in WT mice. **A** Volcano plot displaying the distribution of differentially expressed genes between WT-Sponge and WT-Control. Top 10 DEGs were labeled. **B** Heatmap of DEGs between WT-Sponge and WT-Control. Downregulated DEGs in WT-Sponge vs WT-Control were depicted in blue and upregulated DEGs were in orange. **C** GO enrichment analysis of upregulated DEGs in WT-Sponge. **D** Expression of DEG that is the predicted target of miR-29a

From transcriptomic profiling of WT mice, we found 27 transcripts that differed between control and miR-29a-sponge status at FDR < 0.1. Of the 27 DEGs, 17 were upregulated and 10 were downregulated (Fig. 5A and B, Supplementary Table 3). GO analysis showed the upregulated genes in WT-Sponge were enriched for genes implicated in visual learning, visual behavior, and associative learning (Fig. 5C). Similarly, the upregulated genes in WT-Sponge were predicted miR-29a targets [27]. Plxna1 was found to meet both criteria (Fig. 5D) in WT mice.

We also sought to identify the downstream effectors of miR-29a at the protein level since miRNAs alter gene expression either through mRNA degradation or translation inhibition. Thus, we performed proteomics on hippocampi using the same association testing as was used for the transcriptomic analyses. We imposed a completeness threshold of 50% on the proteins analyzed to limit the influence of less abundant proteins that are more likely to be incompletely sequenced in different samples. A total of 6559 proteins were used for differential expression analysis testing but we found no significant differential proteins for FAD-Sponge vs. FAD-Control or WT-Sponge vs. WT-Control FDR < 0.01 (Supplementary Tables 4–5). Since our goal is to identify the downstream targets of miR-29a in 5×FAD and WT mice, we next selected the proteins that are predicted targets based on the miRDB database [27]. A total of 335 proteins were selected and used for further differential expression analysis. We found Wdfy1 was significantly upregulated in FAD-Sponge compared with FAD-Control at FDR < 0.05 (Fig. 4E and F, Supplementary Table 6–7). Wdfy1 was also found to be up-regulated in FAD-Sponge at the transcript level, suggesting miR-29a could influence learning and memory in 5×FAD mice through WDFY1.

## Discussion

Our prior work identified miR-29a as strongly associated with cognitive decline in humans. To test for a causal relationship between miR-29a and cognitive trajectory, we investigated whether the loss of miR-29a function in mouse brains would influence memory by delivering an AAV-expressing miR-29a sponge to the hippocampus of 5×FAD and WT mice. As secondary analyses, we explored the effect of miR-29a on hippocampal pathology and gene expression. Notably, the administration of the miR-29a sponge to either WT or FAD mice improved some measures of learning and memory. The molecular consequences of lowering miR-29a levels were tested by measuring beta-amyloid deposition in FAD mice and by measuring activated astrocytes and microglia by immunofluorescence staining in WT and FAD. We found that beta-amyloid deposition was reduced in FAD mice receiving the miR-29a sponge, and measures of activated astrocytes and microglia were lower for WT and FAD mice receiving the miR-29a sponge compared to their respective control groups. To identify potential molecular effectors of miR-29a, we profiled gene expression using transcriptomic and proteomic sequencing that identified Plxna1 and Wdfy1 as putative effectors at the transcript and protein level in WT and 5×FAD mice, respectively.

Previous studies showed that miR-29a increases with age across different species and tissues [31–35], and miR-29a regulates age-dependent processes such as neuronal maturation and iron accumulation in the brain [36, 37]. Increased miR-29a may be a normal response to escalating neuroinflammatory load [34]. In line with this notion, our data confirmed that endogenous expression of miR-29a increased with age in WT mice. Interestingly, 2-month-old 5×FAD mice displayed miR-29a levels comparable to that of 12-month-old WT mice, and miR-29a expression remained essentially unchanged over time in the transgenics, raising the possibility of a dysfunctional response to increased brain inflammation in aged 5×FAD mice.

We have previously reported that higher miR-29a levels were associated with faster cognitive decline in older people [5]. Consistent with prior findings, our data showed that miR-29a loss-of-function could improve some measures of learning and memory in both 5×FAD and WT mice. Immunofluorescence staining further indicated that miR-29a sponge attenuated the amyloid deposition and decreased the activated astrocytes and microglia in the mouse hippocampus, highlighting the immunomodulatory role of miR-29a. Transcriptomic and proteomic profiling designed to identify putative causal genes or sets of genes influenced by reduced miR-29a expression revealed different results in the WT and 5×FAD mice, although both showed distinct alterations in genes targeted by miR-29a.

In 5×FAD mice, miR-29a sponge was significantly associated with genes involved in glial cell differentiation and myelination. Among them, PLP1, ERMN, MBP, and MAG are highly expressed in oligodendrocytes and are canonical oligodendrocyte markers [38]. In a recent study, the 5×FAD mouse transcriptomic signatures matched an inflammation-driven clinical AD subtype with an increase of oligodendrocyte and astrocyte markers [39]. We have also previously reported that the decreased abundance of myelination-related proteins such as MBP was associated with cognitive stability in a proteome-wide association study [40]. Among miR-29a target genes in 5×FAD mice, WDFY1 and DIO2 were notably upregulated by expressing miR-29a sponge. Wdfy1 is a phosphatidylinositol 3-phosphate binding protein. Kong et al. reported that Wdfy1 attenuated neuroinflammation [41], and they also found that forsythoside B attenuated memory impairment and neuroinflammation through increased Wdfy1 expression in an AD animal model [41]. WDFY1 and WDFY family were also reported to be involved in neurogenesis, cerebral expansion, and functional organization [42, 43]. In the present study, WDFY1 was found to be upregulated in miR-29 sponge-treated 5×FAD mice at both the transcript and protein level, suggesting it is a likely downstream target of miR-29a. Dio2 influences thyroid hormone action by converting the prohormone thyroxine (T4) to bioactive 3,3′,5-triiodothyronine (T3) [44]. Dio2 was found to be significantly downregulated in AD [45].

By contrast, miR-29a sponge expression in WT mice was associated with changes in genes involved in visual learning, visual behavior, and associative learning. Among predicted targets of miR-29a, Plxna1 was differentially expressed. PLXNA1 is a transmembrane receptor mediating semaphorin signaling [46, 47] and has been reported to regulate the development of neurons and axonogenesis during early development [48, 49]. Plexin signaling is believed to be involved in AD [49]. In our prior work, we also found that higher abundance of PLXNA1 is associated with cognitive stability in a proteome-wide association study of cognitive trajectory [5].

A possible reason why we found different downstream effectors of miR-29a in 5×FAD and WT mice is that the ability of miRNA sponges to derepress target mRNAs depends on the abundance of miRNA, miRNA targets, or both [6]. Here, we find that the endogenous expression of miR-29a differed in the hippocampi of 5×FAD and WT mice, which may underlie why we found different downstream targets in 5×FAD and WT mice. Additionally, target competition effects could also be influenced by small differences in miR-29a sponge injection.

Our findings have limitations and areas that require further exploration in future work. First, the putative downstream effectors were not validated through quantitative PCR or western blotting, which limits our ability to determine false positive results. Second, replication of the immunohistochemistry would be strengthened by replication and testing of additional brain regions. Third, quantification of neuronal loss in the subiculum could explain the protective effect of miR-29a sponge on memory and ought to be explored. Fourth, the miR-29a sponge was only delivered to mouse hippocampi for miR-29a loss of function, and a more widespread lowering of miR-29a is needed. Finally, the molecular mechanism for the protective effect of miR-29a needs careful exploration.

In conclusion, we present evidence from mouse models of AD that miR-29a can modulate the cognitive trajectory that we originally observed in human brains. The effects we observed after the targeted delivery of the miR29a-sponge to the hippocampus on behavior were modest and most evident in 5×FAD mice, which is consistent with the observations in the human brain. Interestingly, we observed that lowering miR-29a reduced beta-amyloid deposition in 5×FAD mice and measures of astrocyte and microglial activation in 5×FAD and WT mice. Transcriptomic and proteomic investigation of mouse brains points to putative targets of miR-29a. These targets differ in 5×FAD and WT mice, possibly related to different baseline levels of miR-29a and cell type composition in these mice. Further study of WDFY1 and PLXNA1 in different cell types would likely shed light on the context that miR-29a acts to ameliorate cognitive decline with age.

### Supplementary Information

ESM 1 (XLSX 7446 kb)

ESM 2 (DOCX 25 kb)

## Data Availability

Upon publication phenotypic, transcriptomic, and proteomic data used in this manuscript will be available at Synapse ID: syn51236902. Supplementary Tables are available on Synapse at syn51369737.
