# Supplementary material for: Lowering Hippocampal miR-29a Expression Slows Cognitive Decline and Reduces Beta-Amyloid Deposition in 5×FAD Mice
Source: Mol Neurobiol. 2023 Nov 22;61(6):3343–56. doi: 10.1007/s12035-023-03791-0 (PMC11087195; doi:10.1007/s12035-023-03791-0)
Supplement: Supplementary file 2 — (DOCX 25 kb) [file 12035_2023_3791_MOESM2_ESM.docx]

**Supplementary Methods**

**Protein digestion and TMT labeling**

Each mouse hippocampus sample was digested individually using the EasyPep^™^ mini sample preparation kit according to manufacturer instructions (ThermoFisher Scientific, A40006). Briefly, each sample was homogenized in 200 uL kit lysis buffer with Halt protease inhibitors and nuclease. A protein concentration assay was performed and 80ug went through digestion and desalting. Resulting peptides were desalted with a Sep-Pak C18 column (Waters, WAT054945) and dried under vacuum. Peptides were reconstituted in 100ul of 100mM triethyl ammonium bicarbonate (TEAB) and labeling performed as previously described^17,18^. One batch of 16-plex TMTPro isobaric tags (Thermofisher Scientific, A44520) was used to label all 16 samples. All 16 channels were then combined and dried by SpeedVac (LabConco) to approximately 100 μL and diluted with 1 mL of 0.1% (vol/vol) TFA, then acidified to a final concentration of 1% (vol/vol) FA and 0.1% (vol/vol) TFA. Peptides were desalted with a 60 mg HLB plate (Waters). The eluates were then dried to completeness.

**High pH Fractionation**

High pH fractionation was performed essentially as described^19^ with slight modification. Dried samples were re-suspended in high pH loading buffer (0.07% vol/vol NH4OH, 0.045% vol/vol FA, 2% vol/vol ACN) and loaded onto a Water’s BEH (2.1mm x 150 mm with 1.7 µm beads). An Thermo Vanquish UPLC system was used to carry out the fractionation. Solvent A consisted of 0.0175% (vol/vol) NH4OH, 0.01125% (vol/vol) FA, and 2% (vol/vol) ACN; solvent B consisted of 0.0175% (vol/vol) NH4OH, 0.01125% (vol/vol) FA, and 90% (vol/vol) ACN. The sample elution was performed over a 25 min gradient with a flow rate of 0.6 mL/min with a gradient from 0 to 50% B. A total of 96 individual equal volume fractions were collected across the gradient and dried to completeness using a vacuum centrifugation.

**Liquid Chromatography Tandem Mass Spectrometry**

All samples (~1ug for each fraction) were loaded and eluted using Dionex Ultimate 3000 RSLCnano (Thermofisher Scientific) an in-house packed 15 cm, 100 μm i.d. capillary column with 1.9 μm Reprosil-Pur C18 beads (Dr. Maisch, Ammerbuch, Germany) using a 23 min gradient. Mass spectrometry was performed with a high-field asymmetric waveform ion mobility spectrometry (FAIMS) Pro equipped Orbitrap Eclipse (Thermo) in positive ion mode using data-dependent acquisition with 2 second top speed cycles. Each cycle consisted of one full MS scan followed by as many MS/MS events that could fit within the given 2 second cycle time limit. MS scans were collected at a resolution of 120,000 (410-1600 m/z range, 4x10^5 AGC, 50 ms maximum ion injection time, FAIMS compensation voltage of -45). All higher energy collision-induced dissociation (HCD) MS/MS spectra were acquired at a resolution of 30,000 (0.7 m/z isolation width, 35% collision energy, 1.25×10^5 AGC target, 54 ms maximum ion time, TurboTMT on). Dynamic exclusion was set to exclude previously sequenced peaks for 20 seconds within a 10-ppm isolation window.

**Protein identification and quantification**

All raw files were searched using Thermo's Proteome Discoverer suite (version 2.4.1.15) with Sequest HT. The spectra were searched against a mouse uniprot database downloaded August 2020 (91414 target sequences). Search parameters included 20ppm precursor mass window, 0.05 Da product mass window, dynamic modifications methione (+15.995 Da), deamidated asparagine and glutamine (+0.984 Da), phosphorylated serine, threonine and tyrosine (+79.966 Da), and static modifications for carbamidomethyl cysteines (+57.021 Da) and N-terminal and Lysine-tagged TMT (+304.207 Da). Percolator was used filter PSMs to 0.1% FDR. Peptides were group using strict parsimony and only razor and unique peptides were used for protein level quantitation. Reporter ions were quantified from MS2 scans using an integration tolerance of 20 ppm with the most confident centroid setting. Only unique and razor (i.e., parsimonious) peptides were considered for quantification.
